# Exploring the Effect of Resveratrol, Tyrosol, and Their Derivatives on Platelet-Activating Factor Biosynthesis in U937 Cells

**DOI:** 10.3390/molecules29225419

**Published:** 2024-11-17

**Authors:** Filio Petsini, Maria Detopoulou, Maria Choleva, Ioannis K. Kostakis, Elizabeth Fragopoulou, Smaragdi Antonopoulou

**Affiliations:** 1 Department of Nutrition and Dietetics, School of Health Sciences and Education, Harokopio University, 70 Eleftheriou Venizelou Avenue Kallithea, 17676 Athens, Greece; fpetsini@hua.gr (F.P.); mdetopoulou@gmail.com (M.D.); mcholeva@hua.gr (M.C.); efragop@hua.gr (E.F.); 2 Department of Pharmacy, Division of Pharmaceutical Chemistry, National and Kapodistrian University of Athens, 15771 Athens, Greece; ikkostakis@pharm.uoa.gr

**Keywords:** cholinephosphotransferase, acetyltransferase, phenolic compounds, interleukin-1β, inhibitors, inflammation, messaging pathways, extracellular signal-regulated kinase, mitogen-activated protein kinase, phospholipase C-β

## Abstract

Platelet-activating factor (PAF) is a potent lipid mediator, involved in thrombosis, inflammation, and atherosclerosis. The protective effect of wine and olive oil against atherosclerotic diseases is largely attributed to their phenolic compounds and mostly to resveratrol and tyrosol. Both compounds have been reported to inhibit PAF biosynthesis in interleukin-1β (IL-1β)-stimulated monocytes and also to attenuate PAF biosynthesis in cell lysates. The aim of this study was to investigate the effects of resveratrol, tyrosol, and their derivatives on unstimulated U937 cells and to explore the intracellular messaging pathways that participate in the activation of PAF biosynthesis in the same cell line. Tyrosol and its derivatives did not exert any substantial effect on PAF biosynthesis. Resveratrol (50 and 100 μM), as well as its methoxy derivative (5–20 μM), caused a reduction in the PAF biosynthetic enzymes’ activity by 20–43% after 24 h of incubation. On the other hand, lower resveratrol concentration (10 μM) and higher concentration of the methoxy derivative (50 μM) increased the Ca^2+^-dependent lyso–PAF acetyltransferase (LysoPAF-ATC) activity by 28–45% after half-hour incubation via p38 mitogen-activated protein kinase (p38-MAPK) action. IL-1β activated PAF biosynthetic pathways via different signaling pathways, with phospholipase C-β (PLC-β) being a key enzyme.

## 1. Introduction

In recent decades, in the research community, there has been an ever-growing interest in the bioactive compounds present in foods. A great number of bioactive compounds have been reported: fish contain n-3 polyunsaturated fatty acids (PUFA) such as eicosapentaenoic acid (EPA) [1], whereas olive oil mainly contains the monounsaturated (MUFA) oleic acid [2], along with other polar lipids [3], lipid minor constituents [4], and phenolic components [5]. Bioactive peptides in yogurt and cheese are products of protein fermentation [6,7]; eggs are rich in vitamins and choline [8]; β-carotene-a phenolic vitamin A precursor is found in vegetables [9], among other vitamins and polyphenols; whole-grain cereals are an important source of β-glucan, lignans, and dietary fiber [10,11]; and anthocyanins are flavonoids that also act as pigments for fruits from red to blue-violet colors [12]. The list is endless. To this diverse group of food components are attributed health-beneficial properties, spanning from cardiovascular and inflammatory protection to anticancer and neuroprotective abilities [1,4,7,10,13,14,15,16]. Balanced diets, like the Mediterranean diet, are characterized by a plethora of foods that contain these micro-constituents; for example, olive oil, wine, vegetables, and fruits contain members of the wide family of phenolic compounds. Phenolic compounds present antioxidant, anti-inflammatory and antithrombotic abilities [12,17,18,19], among others, and therefore attract scientific interest when it comes to their effect on cardiovascular health. According to the Prevención con Dieta Mediterránea (PREDIMED) study [20], frequent polyphenol intake is inversely correlated with the risk for major cardiovascular incidents.

Resveratrol, a stilbene primarily detected in grapes and wine, exhibits cardioprotective, anti-inflammatory and anticancer properties, and it is well investigated in the literature, from in vitro studies to clinical trials [21,22,23,24]. It is reported that this molecule can act through various mechanisms in different cells [25], and most importantly, it exhibits a dual action as it can either inhibit or stimulate the same messaging pathways [26]. For instance, resveratrol concentrations lower than 10 μM induce activation of extracellular signal-regulated kinases 1 and 2 (ERK1/2) and mitogen-activated protein kinases (MAPK) [27,28], whereas concentrations higher than 50 μM inhibit ERK1/2 and MAPK [27,29]. Although the data are sparse, resveratrol has also been found to promote inflammation in lipopolysaccharide (LPS)-stimulated human leukocytes [30] and stimulated murine vascular smooth muscle cells [31]. Nevertheless, since resveratrol is quickly metabolized by the human body and therefore of poor bioavailability, research has also been focusing on more stable derivatives as a way of improving the action of the molecule [32].

Tyrosol is one of the major phenolic compounds of olive oil, along with oleuropein and hydroxytyrosol [33]. This rather simple molecule is known to protect against oxidation and inflammation and to exert an antiatherogenic effect [34]. Tyrosol is capable of affecting messaging pathways as it suspends LPS-induced MAPK, ERK, and nuclear factor kappa-Β (NF-κB) activation in RAW 264.7 macrophage cells [35,36].

Platelet-activating factor (PAF), a potent phospholipid mediator, is considered to have a significant role to the appearance and development of cardiovascular diseases (CVDs) [37]. It is produced by a variety of cells—for instance, platelets, monocytes, and endothelial cells—which are also related to inflammation and thrombosis. These defensive mechanisms can lead to serious implications when they are not properly controlled by the body, and PAF seems to be a key factor in these situations [38,39]. Both PAF metabolism and PAF actions through its receptor (PAFR) can be regulated or inhibited by bioactive compounds present in foods in the Mediterranean diet, especially in fish, olive oil, and wine [40,41,42]; for example, red wine can suppress PAF biosynthesis postprandially [43], whereas PAF metabolism is reduced after long-term consumption of yogurt enriched with an olive oil by-products extract, rich in PAF inhibitors [44].

**Figure 1:**
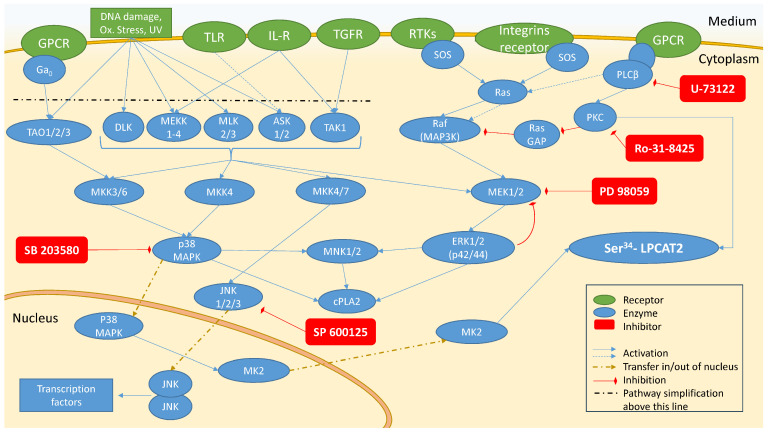
Presentation of the messaging pathways of mitogen-activated protein kinases (MAPK), c-Jun N-terminal kinase (JNK), extracellular signal-regulated kinases (ERK), protein kinase C (PKC), and phospholipase C-β (PLC-β). Pathways are simplified upstream of the dashed line to facilitate interpretation. cPLA2, cytosolic phospholipase A_2_; GPCR, G-coupled proteins receptor; IL-R, interleukins receptor; LPCAT2, lysophosphatidylcholine acyltransferase 2; MEK 1/2, kinases of ERK1/2; MK2, MAPK-activated kinase 2; MKK3,4,6,7, kinases of p38 MAPK; TLR; lipopolysacharide (LPS) receptor.

PAF is produced both constitutively and under inflammatory conditions via two distinct pathways. De novo biosynthesis of PAF occurs mainly under basal conditions, and the key enzyme of the final reaction is 1-alkyl-2-acetyl-*sn*-glycerol cholinephosphotransferase (PAF-CPT) [45]. The remodeling pathway is regulated by acetyl-coenzyme A: lyso–platelet-activating factor acetyltransferases (LysoPAF-AT), and two isoforms of this enzyme have been identified to date. One is activated during inflammation requiring the presence of Ca^2+^ (LPCAT2 or LysoPAF-ATC) [46], while the second one is calcium and inflammation-independent (LPCAT1 or LysoPAF-ATE) [47]. LPCAT2 is the most investigated of the three biosynthetic enzymes [48]. It belongs to the greater family of lysophospholipid acyltransferases, along with LPCAT1, and is activated by phosphorylation. LPS induces inflammation and LPCAT2 activation through toll-like receptor 4 (TLR4), activating the messaging cascades of MAPK [49]. Protein kinase C (PKC) and phospholipase C-β (PLC-β) can also activate LPCAT2 when macrophages are stimulated through G protein-coupled receptors (GPCRs), such as PAFR [50]. LPCAT1 biosynthesizes PAF under noninflammatory conditions and is mainly present in lung microsomes [47]. PAF-CPT is located in the endoplasmic reticulum (ER), but research is scarce regarding its activating pathways [51], and so far, any isolation of the enzyme has failed as the enzyme loses its function when it is separated from the membranes [52,53]. Interleukin 1β (IL-1β) is known to promote PAF biosynthesis through both the de novo and the remodeling pathways in U937 cells [54]. IL-1β binding to its receptor can ignite messaging pathways that include p38 MAPK, ERK, and c-Jun N-terminal kinases (JNK) [55], kinases interlaced with the phosphorylation of LPCAT2 [49,56,57]. A graphical interpretation of the various kinases that may regulate PAF biosynthesis by LPCAT2, according to literature, along with their inhibitors, is presented in Figure 1; to our knowledge, there are no data of potential messaging pathways related to PAF-CPT.

Resveratrol, tyrosol, and their acetylated and methylated derivatives can inhibit PAF-induced platelet aggregation [58,59]. Resveratrol is also capable of inhibiting PAF-CPT or LysoPAF-AT activity in both intact and homogenized U937 cells [60], and a similar or more potent action was observed for a methylated and an acetylated derivative regarding homogenized U937 cells [61]. Tyrosol and two acetylated derivatives exhibited a less potent inhibitory effect on PAF biosynthetic enzymes activity. Resveratrol, tyrosol, and their derivatives can also suppress the activity of PAF-CPT and LysoPAF-AT in IL-1β-stimulated U937 cells [62,63]. Among the derivatives, 3-methoxyresveratrol (RMeth2) and 3,5-diacetylresveratrol (RAc3), tyrosol acetylated at the aliphatic hydroxyl group (–OH) (TAc2), and diacetylated tyrosol (TAc3) displayed the most potent inhibiting effect on PAF biosynthesis, similarly to the parent compounds. These derivatives were chosen to be examined in the present study.

The scope of this study was to explore the messaging pathways that participate in the activation of PAF-CPT and LysoPAF-AT under inflammatory conditions in U937 cells. The effect of resveratrol and its derivatives on PAF biosynthesis in U937 cells was also examined after 30 min of incubation, linking this effect to signaling pathways. Last but not least, U937 cells were incubated with the resveratrol, tyrosol, and their derivatives for 24 h in order to explore whether these phenolic compounds can interfere with translational pathways under basal conditions.

## 2. Results

### 2.1. Effect of Phenolic Compounds, of Their Derivatives, and of Signal Transduction Inhibitors on Cell Viability

Initially, the effect of all phenolic compounds, as well as the signal transduction inhibitors, on cells viability was tested using the thiazolyl blue tetrazolium bromide (MTT) assay, and the accepted level of viability was over 80% (Table 1) [64]. In particular, the concentration of phenolic compounds ranged from 10 to 100 μΜ for resveratrol and its derivatives and from 0.1 to 100 μM for tyrosol and its derivatives. The tested concentration range of inhibitors varied for each inhibitor but, in general, it was between 0.25 and 100 μM. Results revealed that resveratrol and its derivatives were non-toxic at 100 μΜ for short-time incubation, but in long-term incubation, RMeth2 could be used at a maximum of 20 μΜ and RAc3 at a maximum of 75 μM. Tyrosol was not toxic at 100 μM, and its derivatives were not toxic in the range between 0.1 and 5 μM; no higher concentration was tested. Inhibitors were assayed in the following concentrations: SB203580 20 μΜ, PD 98059 25 μM, U-73122 1.5 μM, RO-31-8425 2.5 μM, and SP 600125 5 μM.

### 2.2. Investigation of Signal Transduction Involved in Activation of PAF Biosynthetic Enzymes in U937 Cells

#### 2.2.1. Enzymes’ Activity in Unstimulated Cells (Basal Conditions) and IL-1β-Stimulated Cells

**Figure 2:**
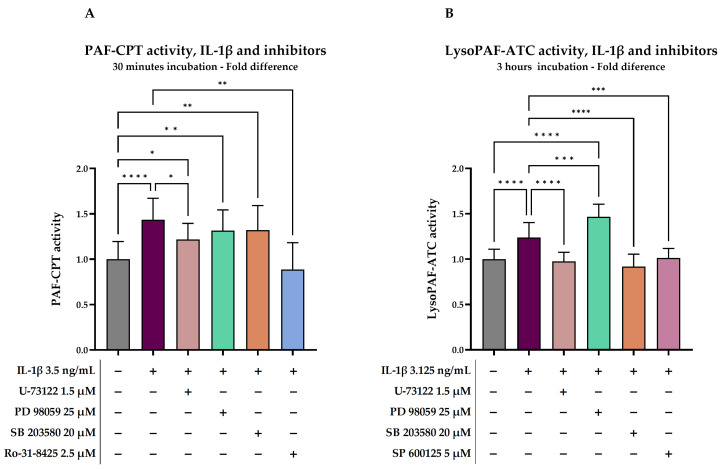
(**A**) PAF-CPT activity in IL-1β-stimulated U937 cells and the effect of inhibitors after 30 min of incubation. (**B**) LysoPAF-ATC activity in IL-1β-stimulated U937 cells and the effect of the inhibitors after 3 h of incubation. Different colors represent different inhibitors. Results are expressed as a fold difference of the baseline levels of enzymatic activity. Statistically significant differences are expressed as follows: (*) when *p*-value < 0.05, (**) when *p*-value < 0.01, (***) when *p*-value < 0.001, and (****) when *p*-value < 0.0001. IL-1β, interleukin 1β; LysoPAF-ATC, Ca^2+^ dependent lysoPAF acetyltransferase; PAF, platelet activating factor; PAF-CPT, 1-alkyl-2-acetyl-sn-glycerol cholinephosphotransferase.

The activity of PAF-CPT and LysoPAF-ATC in unstimulated cells was 0.649 ± 0.177 pmol PAF/min/μg protein and 0.708 ± 0.166 pmol PAF/min/μg protein, respectively. IL-1β increased PAF-CPT activity by 43.6% after 30 min of incubation (Figure 2A, Table 2). IL-1β stimulated LysoPAF-ATC activation by 23.8% after 3 h of incubation (Figure 2B, Table 3).

#### 2.2.2. Investigation of Signal Transduction on IL-1β-Stimulated Cells

In order to investigate the signal transduction pathways that occurred during activation of PAF biosynthetic enzymes by IL-1β, U937 cells were pre-treated with signal transduction inhibitors. The effect of IL-1β on PAF-CPT activity was completely inhibited by Ro-31-8425, a selective inhibitor of PKC (*p* = 0.0017). In the presence of U-73122, a PLC-β inhibitor, IL-1β, was able to stimulate the PAF-CPT activity only by 21.8% compared to baseline (*p* =0.0165), 50% less than when IL-1β acted alone (*p* = 0.0155). SB 203580 (p38 MAPK inhibitor) and PD 98059 (MEK 1/2—kinases of ERK1/2—inhibitor) were not able to attenuate the effect of IL-1β (Figure 2A).

U-73122, SB 203580, and SP 600125 (JNK inhibitor) were all able to completely inhibit IL-1β stimulation of LysoPAF-ATC (*p* = 0.0006, *p* < 0.0001, and *p* = 0.0006, respectively). On the other hand, the simultaneous presence of IL-1β and PD 98059 enhanced LysoPAF-ATC activity by 46.7% compared to baseline (*p* < 0.0001), reaching 1.004 ± 0.225 pmol PAF/min/μg protein, and almost doubling the effect of IL-1β alone (*p* < 0.0001) (Figure 2B).

### 2.3. Short-Time Effect of Resveratrol and Its Derivatives on PAF-CPT and LysoPAF-ATC in U937 Cells

We previously reported [61] that resveratrol, RMeth2, and RAc3 can attenuate the activity of PAF-CPT and LysoPAF-ATC in cell lysates. In the present study, an experiment was carried out in order to observe whether the phenolic compounds may also affect the messaging pathways that control the biosynthesis of PAF in U937 cells under basal conditions.

#### 2.3.1. Enzymes’ Activity in Unstimulated Cells (Basal Conditions)

After 30 min of incubation under basal conditions, PAF-CPT, the main enzyme for PAF production under basal conditions, displayed an activity of 1.304 ± 0.06 pmol PAF/min/μg protein, while LysoPAF-ATC displayed an activity of 0.483 ± 0.134 pmol PAF/min/μg protein.

#### 2.3.2. Effect of Resveratrol and Derivatives on PAF-CPT and LysoPAF-ATC

**Figure 3:**
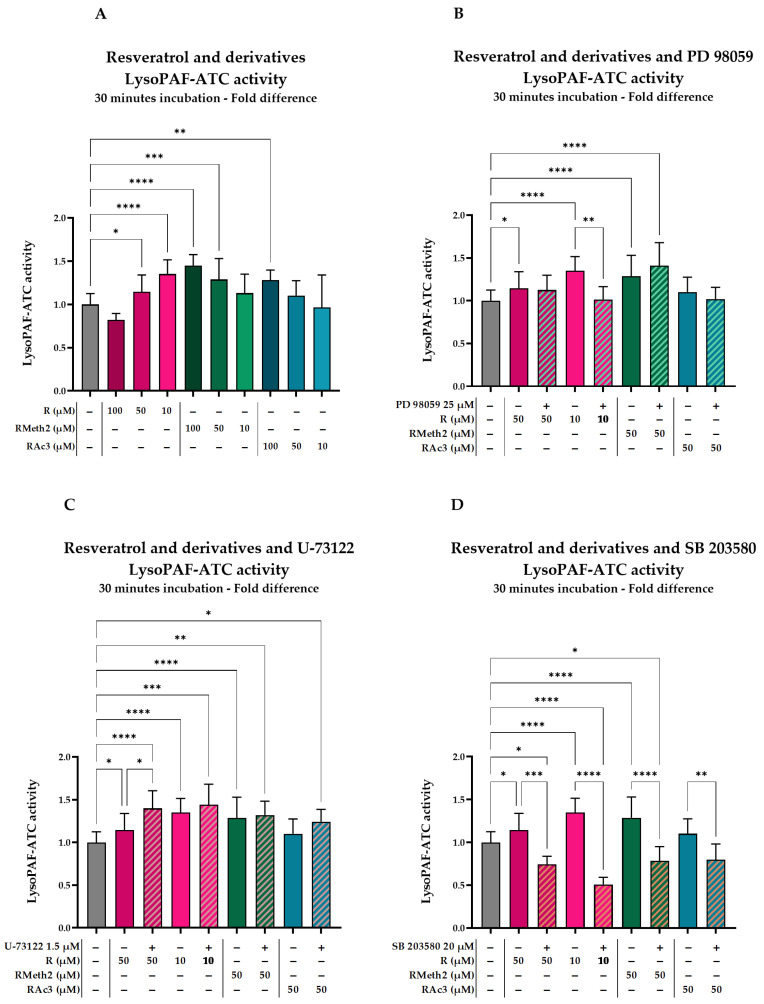
(**A**) Effect of resveratrol and its derivatives on LysoPAF-ATC after 30 min of incubation. (**B**) Effect of resveratrol and its derivatives on LysoPAF-ATC after 30 min, with and without PD 98059. (**C**) Effect of resveratrol and its derivatives on LysoPAF-ATC after 30 min, with and without U-73122. (**D**) Effect of resveratrol and its derivatives on LysoPAF-ATC after 30 min, with and without SB 203580. Different color families represent different compounds and different shades of the same color family represent different concentrations of the same compound; the darker the shade the higher the concentration. Single colored bars represent results for a compound alone and striped bars represent results with the presence of the respective inhibitor. Results are expressed as a fold difference of the baseline levels of enzymatic activity. Statistically significant differences are expressed as follows: (*) when *p*-value < 0.05, (**) when *p*-value < 0.01, (***) when *p*-value < 0.001, and (****) when *p*-value < 0.0001. R, resveratrol; RMeth2, 3-methoxyresveratrol; RAc3, 3,5-diacetylresveratrol.

De novo biosynthesis by PAF-CPT remained unaffected after 30 min incubation (Table 4) for all different compounds in several concentrations. Regarding LysoPAF-ATC, resveratrol enhanced PAF biosynthesis in a reverse dose-dependent manner, with 10 μM resulting in 35.0% higher LysoPAF-ATC activity (*p* < 0.0001), whereas 100 μM resveratrol resulted in a non-significant drop in activity (Figure 3A, Table 4). The opposite behavior was observed in the derivatives where higher concentrations led to greater LysoPAF-ATC activation (Figure 3A); 100 μM of RMeth2 and RAc3 increased enzymatic activity by 44.8 and 28.0%, respectively (*p* < 0.0001 and *p* = 0.0058, respectively). Between the two derivatives, RMeth2 had a stronger effect since 50 μM also activated LysoPAF-ATC by 28.7% (*p* = 0.0001).

#### 2.3.3. Effect of Inhibitors on the LysoPAF-ATC Activation by Resveratrol and Its Derivatives in U937 Cells After 30 min

In Section 2.2, we determined that the activation of LysoPAF-ATC by IL-1β was greatly hindered when inhibitors of p38 MAPK (SB 203580), JNK (SP 600125), and PLC-β (U-73122) were present, whereas MEK 1/2 inhibitor (PD 98059) further increased the LysoPAF-ATC activation. The most potent inhibitors of IL-1β action (SB 203580 and U-73122), along with PD 98059, which increased its action, were selected to be tested in combination with the phenolic compounds.

PD 98059 completely inhibited the LysoPAF-ATC activation caused by 10 μM resveratrol (*p* = 0.0037) (Figure 3B, Table 5), whereas it had no significant effect on LysoPAF-ATC activation induced by 50 μΜ RMeth2 and RAc3, respectively (Figure 3B, Table 5).

The stimulatory effect of 50 μΜ resveratrol was further enhanced by an extra 22.2%, in the presence of the U-73122 inhibitor (*p* = 0.0127) (Figure 3C, Table 6). Pre-incubation with U-73122 did not affect LysoPAF-ATC activation induced by RMeth2 and RAc3 (Figure 3C, Table 6).

The presence of the SB 203580 inhibitor completely inhibited LysoPAF-ATC activation induced by all three phenolic compounds (Figure 3D, Table 7). The inhibition was more profound in the case of LysoPAF-ATC activation induced by 10 μM resveratrol (*p* < 0.0001), where the enzymatic activity was halved compared to baseline (Table 7).

### 2.4. Long-Term Effect of Resveratrol, Tyrosol, and Their Derivatives on PAF-CPT, LysoPAF-ATC and LysoPAF-ATE

#### 2.4.1. Enzymes’ Activity in Unstimulated Cells (Basal Conditions)

After 24 h of incubation, PAF-CPT displayed an activity of 0.853 (0.711, 1.205) pmol PAF/min/μg protein, LysoPAF-ATC activity was 0.351 (0.310, 0.387) pmol PAF/ min/μg protein, and LysoPAF-ATE activity was 0.054 (0.040, 0.065) pmol PAF/ min/μg protein.

#### 2.4.2. Effect of Resveratrol and Derivatives on Enzymes

Resveratrol was initially tested in a wide range of concentrations, from 0.1 to 100 μM, and its effective concentration area was determined to be between 10 and 100 μM (Table 8). The maximum concentrations for RMeth2 and RAc3 tested were 20 and 75 μM, respectively, based on the results from the viability assay.

**Figure 4:**
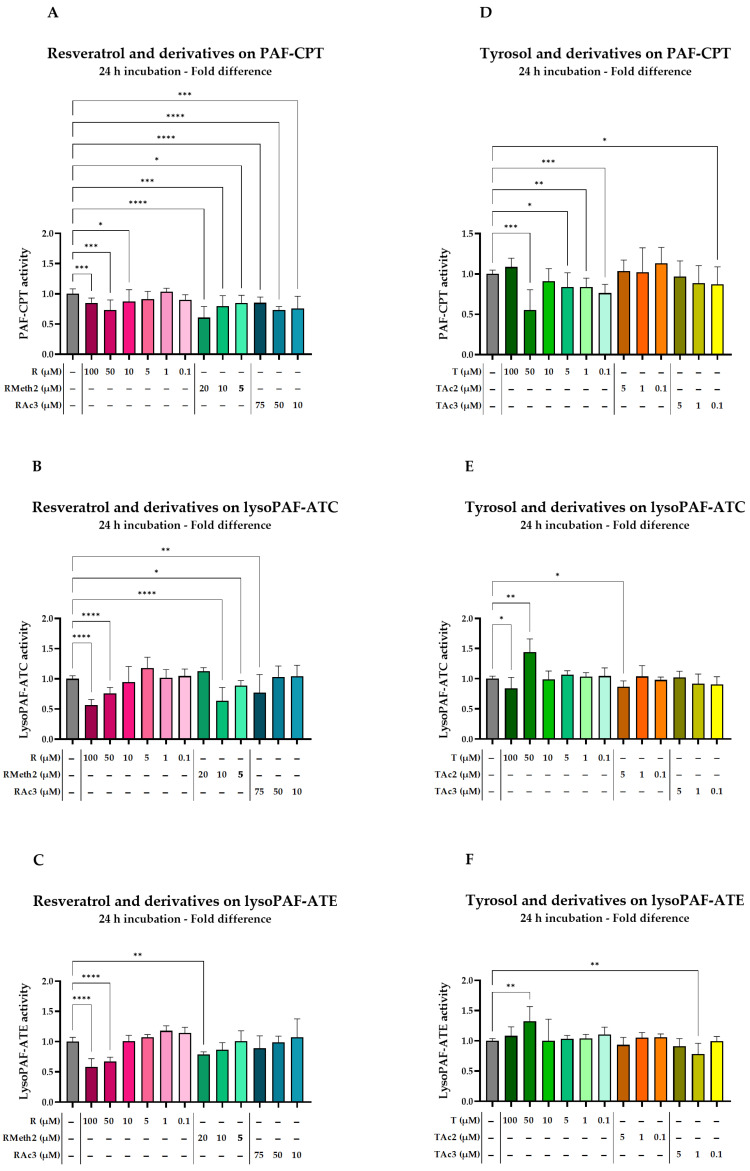
Effect of resveratrol, and derivatives on PAF-CPT (**A**), LysoPAF-ATC (**B**) and LysoPAF-ATE (**C**) activity in U937 cells after 24 h. Effect of tyrosol, and derivatives on PAF-CPT (**D**), LysoPAF-ATC (**E**) and LysoPAF-ATE (**F**) activity in U937 cells after 24 h. Different color families represent different compounds and different shades of the same color family represent different concentrations of the same compound; the darker the shade the higher the concentration. Results are expressed as a fold difference of the baseline levels of enzymatic activity. Statistically significant differences are expressed as follows: (*) when *p*-value < 0.05, (**) when *p*-value < 0.01, (***) when *p*-value < 0.001, and (****) when *p*-value < 0.0001. T, tyrosol; TAc2, tyrosol acetylated at the aliphatic hydroxyl group (–OH); TAc3, diacetylated tyrosol.

In general, resveratrol and its derivatives had the ability to partially inhibit PAF-CPT, but only RMeth2 followed a dose-dependent action (Figure 4A, Table 8). Nevertheless, for the concentrations tested, the inhibition effect did not surpass 39.3% of 20 μM of RMeth2 (*p* < 0.0001), and 50 μM of resveratrol and RAc3 behaved similarly, with a peak inhibition of PAF-CPT at 28.4% (*p* = 0.0005) and 27.0% (*p* < 0.0001), respectively.

Regarding LysoPAF-ATC (Figure 4B, Table 8), RMeth2 was the most potent inhibitor, with 10 μM of the phenolic causing a reduction in the enzyme activity of 36.3% (*p* < 0.0001). Resveratrol exhibited a similar effect only when the concentration reached 100 μM (43.4%, *p* < 0.0001), while 75 μM RAc3 inhibited LysoPAF-ATC by 23.0% (*p* = 0.0028). However, only resveratrol exhibited a dose-dependent inhibition.

Resveratrol inhibited LysoPAF-ATE by 41.6% at 100 μM (*p* < 0.0001), while RMeth2 presented a 21.0% inhibition at 20 μM (*p* = 0.0035) and RAc3 had no significant effect (Figure 4C, Table 8).

#### 2.4.3. Effect of Tyrosol and Derivatives on Enzymes

Tyrosol was initially tested in a wide range of concentrations from 0.1 to 100 μM, and its effective concentration area was determined to be mostly between 0.1 and 5 μM (Table 9), mainly due to a more consistent attenuating effect on PAF-CPT; hence, these concentrations were tested for the derivatives as well. Incubation of U937 cells with tyrosol and derivatives was quite complicated as the results did not follow a dose-dependent pattern.

Tyrosol’s effect on PAF-CPT activity was more consistent in lower concentrations (0.1–5 μM), where it exhibited a mild inhibition of 16 to 24% (Figure 4D, Table 9). The effect of 50 μM of tyrosol should also be mentioned as it resulted in 44.7% (*p* = 0.0005) inhibition of PAF-CPT. The derivatives did not seem to have a significant effect on PAF-CPT activity, with the exception of 0.1 μM of TAc3, which reduced the enzyme activity by 13.0% (*p* = 0.0491).

Regarding LysoPAF-ATC (Figure 4E, Table 9), 50 μM of tyrosol enhanced the enzymatic activity by 44.1% (*p* = 0.002), whereas 100 μΜ of tyrosol resulted in the enzyme’s inhibition by 11.8% (*p* = 0.0193). Lastly, a slight inhibition (13.6%) was also observed with 5 μM TAc2 (*p* = 0.0179).

LysoPAF-ATE was mildly enhanced by the higher concentrations of tyrosol at a maximum of 32.4% (*p* = 0.003) in the presence of 50 μM of the phenolic compound (Figure 4F, Table 9); other than that, only 1 μM of TAc3 showed a significant inhibition of the enzyme’s activity (21.8%, *p* = 0.0027).

## 3. Discussion

The scope of the present study was to investigate the effect of resveratrol, tyrosol, and their derivatives on unstimulated U937 cells and to explore the intracellular messaging pathways that participate in the activation of PAF biosynthesis in the same cell line. In order to select specific signaling pathways, IL-1β was used as an inflammatory mediator since it has previously been shown to stimulate PAF biosynthetic enzymes [54]. In IL-1β-stimulated cells, PAF-CPT, the key enzyme in PAF de novo biosynthesis, was primarily regulated by PKC and secondarily by PLC-β. LysoPAF-ATC, an enzyme known to be up-regulated under inflammatory conditions, seems to be activated by IL-1β mainly through PLC-β, p38 MAPK, and JNK cascades, whereas the ERK1/2 pathway had a more complicated relationship to the enzymes’ activation. Nevertheless, LysoPAF-ATC stimulation by resveratrol and its derivatives, in short-time experiments, was abolished by p38 MAPK inhibitor. Last but not least, long-time incubation with resveratrol and its derivatives led to moderate attenuation of PAF biosynthetic enzymes activities, while tyrosol and its derivatives did not exert any substantial effect on PAF biosynthesis, with the exception of 50 μM of tyrosol, which resulted in 44.4% inhibition of PAF-CPT.

Inflammation is a complicated condition related to a variety of diseases, such as cardiovascular and autoimmune diseases. PAF is implicated in these diseases as a pro-inflammatory mediator, acting through its receptor on the producing cell or neighboring cells and activating the messaging cascades of p38 MAPK, ERK, and JNK [45,65]. LPS and IL-1β activate very similar messaging cascades through their receptors, which have an identical intracellular domain: toll-interleukin-1 receptor (TIR) homology domain (TIR) [66,67]. For this study, IL-1β and human monocytes were chosen over LPS and other cell lines as they provide a model closer to the body-induced inflammation than microbial products. We have already demonstrated that U937 cells stimulated by IL-1β resulted in an increased PAF biosynthesis through activation of both PAF-CPT and LysoPAF-AT [54].

The existing literature on the activation mechanism of PAF-CPT is very limited as de novo biosynthesis of PAF has not been associated with inflammatory conditions, mainly because the enzyme has not yet been isolated. Nevertheless, there is growing evidence that PAF-CPT activity is increased in chronic inflammatory conditions [68], making de novo biosynthesis a new target to regulate. PAF-CPT is located in the ER, and IL-1β can increase its activity by 50% in U937 cells within 30 min [54]. The results of the present study agree with the PAF-CPT activation by IL-1β and expand that knowledge by showing that this action is mediated through PKC and PLC-β. Both PKC and PLC-β are in the messaging cascade regulated by GPCRs, one of which is PAFR, and both kinases can also be activated by PAF [69,70]. These results indicate that PAF-CPT acute activation is probably regulated by enzymes downstream of PKC and PLC-β, but it is unknown whether this can be also provoked by PAF through a positive feedback loop. On the other hand, p38 and p42/44 kinases, which are known to be activated by IL-1β and LPS through TLRs in acute inflammation, did not relate to PAF-CPT activation in the present study. These results represent a first step towards further understanding how PAF-CPT is regulated in cells.

LysoPAF-ATC, also known as LPCAT2, has been the center of attention regarding PAF biosynthesis under inflammatory conditions, and the signaling cascades involved in the enzyme’s activation are well studied. Tumor necrosis factor-alpha (TNF-α), calcium ionophore A23187, and N-formyl-methionyl-leucyl-phenylalanine (fMLP) stimulate LysoPAF-AT through the p38 MAPK messaging pathway in human neutrophils, whereas the ERK pathway (MEK 1/2) seems to have an auxiliary role in LysoPAF-AT phosphorylation through cytosolic phospholipase A_2_ (c-PLA_2_) activation [56,57]. Stimulation of mouse macrophages with LPS for 30 min increased LPCAT2 activity four-fold via p38 MAPK [49]. In the present study, the enhanced LysoPAF-ATC activity induced by IL-1β seems to be mediated, at least in part, by p38 MAPK and PLC-β, which is in accordance with the known dependence of LysoPAF-ATC phosphorylation on PLC-β, especially in acute inflammatory conditions [50]. JNKs are also activated by IL-1β through a similar cascade as p38 [55], and according to our results, they are also involved in LysoPAF-ATC activation, though to a lesser extent than p38 and PLC-β. On the other hand, when a MEK 1/2 inhibitor was used, the stimulation effect of IL-1β was enhanced, contrary to non-significant results in the literature [56,57]. It has been reported that the PD 98059 inhibitor of MEK 1/2 can up-regulate the phosphorylation of p38 MAPK, whereas the SB 203580 inhibitor of p38 MAPK activates ERK downstream of MEK 1/2, leading scientists to consider the possibility that there is regulating cross-talk between the two kinases [71,72]. With respect to this hypothesis, it is possible that we also observed an indirect activation of p38 MAPK in the presence of PD 980959, which strengthens the idea that LysoPAF-ATC is activated through the p38 MAPK cascade, but not through ERK 1/2, under IL-1β stimulation of U937 cells. It has also been reported that PD 98059 may be implicated in reducing Ca^2+^ entry into cells, an effect that is not dependent on its ability to inhibit ERK1/2 [73]. Since PD 98059 has been reported to exert off-target effects, the above results are not so easy to interpret. Further experiments are needed to clarify the exact mechanism.

Resveratrol is mainly known as an anti-oxidant and anti-inflammatory compound, although a pro-inflammatory effect has also been observed [26,27,74,75]. Resveratrol can either attenuate or promote the phosphorylation of messaging proteins, and this dual action of resveratrol seems to be cell and concentration specific. For instance, resveratrol can induce apoptosis in metastatic cancer cells by activating ERK 1/2 [76]. On the other hand, resveratrol can inhibit LPS-induced NF-κB stimulation in macrophages through p38 MAPK and JNK attenuation without affecting ERK 1/2 [77]. Migration of osteoblasts caused by epidermal growth factor (EGF) was inhibited in the presence of resveratrol through the regulation of JNK but not p38 MAPK or ERK 1/2 [78]. Taking into consideration the research on resveratrol action presented above, one may conclude that resveratrol behavior varies a lot among different cells and stimulators. It is also well established that resveratrol activates adenosine monophosphate (AMP)-activated protein kinase (AMPK) [25,79], which, in turn, can protect against inflammation by inhibiting p38 MAPK, ERK, and JNK [80,81]. Little is known regarding the derivatives that were tested in the present study. The derivative 3-methoxyresveratrol exerts a stronger anti-proliferative activity than resveratrol and other methylated derivatives against cancer cells (half-maximal inhibitory concentration—IC_50_ < 70 μM), as well as a more efficient inhibition of thrombin receptor activating peptide (TRAP)-induced platelet aggregation [59]. Both resveratrol and 3-methoxyresveratrol, also known as pinostilbene, enhance retinoid acid-induced differentiation of U937 cells to macrophages, aiding phagocytosis through gene upregulation [82]. 3-methoxyresveratrol is able to activate p38 and ERK 1/2 pathways and prevent oral cancer cell metastasis [83]. 3,5-diacetylresveratrol has the ability to attenuate PAF-induced platelet aggregation to the same level as the parent compound [58].

When U937 cells were treated with resveratrol and its derivatives for a short time (30 min), a picture of acute inflammation was observed: PAF-CPT was not affected but LysoPAF-ATC was significantly activated by the parent compound and the derivatives. In a previous study, PAF-CPT activity was increased after 30 min only when resveratrol was as high as 300 μM; LysoPAF-AT was not affected after a 3 h incubation with 50 μM resveratrol, even though PAF levels were elevated [63], allowing for the hypothesis that the enzyme may have already been activated and downregulated in this time frame. It can clearly be concluded that the phenolic compounds do not act directly on the enzymes—as was observed in experiments on cell lysates [61], where both enzymes were inhibited—but activate signaling cascades related to inflammation. Resveratrol enhanced PAF biosynthesis in a reverse dose-dependent manner, resembling the dual action that has already been reported; lower concentrations of resveratrol enhanced IL-1β-induced PAF biosynthesis, while higher concentrations attenuated the stimulation [63]. On the other hand, the derivatives provided a dose-dependent activation of LysoPAF-ATC, with 3-methoxyresveratrol being the most effective at 50 and 100 μM. These concentrations did not exhibit an apoptosis effect in the viability assay; however, it is possible that messaging cascades are activated, also resulting in LysoPAF-ATC phosphorylation.

According to our findings, SB 203580, a p38 MAPK inhibitor, was capable of repressing the action of resveratrol and its derivatives in inducing LysoPAF-ATC activity. Remarkably, in the case of 10 μM resveratrol, the 35% activation was reversed to a 50% inhibition. This significant effect indicates that p38-MAPK is strongly involved in this pro-inflammatory action of resveratrol and its derivatives. Although PLC-β and the ERK 1/2 pathway seem to be involved in the action of resveratrol and its derivatives, the results were unclear and need to be studied further.

The literature on the anti-inflammatory effects of resveratrol is abundant, providing a variety of protocols with different cell lines, stimulating factors, and resveratrol concentration and incubation time. In LPS-stimulated U937 cells, 15 μΜ of resveratrol for 4 h can downregulate genes related to inflammation [84]. Resveratrol is also known to inhibit vascular endothelial growth factor (VEGF)-induced PAF biosynthesis by LysoPAF-AT in Kaposi’s sarcoma cells [85]. The effect of resveratrol on non-stimulated cells has also been studied. Both LysoPAF-AT and PAF-CPT were inhibited by 50% after 24 h of U937 cells incubation with resveratrol [60]; IC_50_ values were 90.8 and 0.12 μM, respectively.

In the present study, however, the inhibition of PAF-CPT after 24 h of incubation did not exceed 30% even at a resveratrol concentration of 100 μM. The LysoPAF-ATC and LysoPAF-ATE inhibition observed in this study is consistent with the existing literature, since 100 μM of resveratrol achieved a 42% decrease in the enzyme’s activity. 3-methoxyresveratrol was able to achieve a similar percentage of inhibition for both enzymes with lower concentrations but without exceeding the 50% threshold; higher concentrations were cytotoxic and therefore could not be assayed. Acetylation resulted in a less potent molecule, which indicates that not only substitution but also the nature of the substitute group affects the behavior of resveratrol derivatives. When comparing the phenolics’ effect on LysoPAF-ATC and LysoPAF-ATE, resveratrol and 3,5-diacetylresveratrol do not demonstrate selectivity between the two enzymes. In the case of 3-methoxyresveratrol, the inhibition of LysoPAF-ATC was more profound at 10 μM, which suggests that the methylated derivative may be a more potent inhibitor of PAF-ATC. A similar result has already been observed for N-phenylmaleimide derivatives that provided a selective inhibition of LPCAT2 over LPCAT1 [86]. 3-methoxyresveratrol has also been identified as a major metabolite of 3,5-dimethoxyresveratrol or pterostilbene, exerting anticancer properties [87].

Tyrosol is less studied regarding its mechanism of action due to its fast clearance in vivo and the high popularity of its sibling, hydroxytyrosol. Nevertheless, tyrosol is reported to inhibit inflammatory response following LPS-induced inflammation in macrophages [35,88], intestinal cells [89], and murine models [36,90], with p38 MAPK, ERK 1/2,and NF-κB regulation being possible points of action. The antioxidative and anti-inflammatory activity of 100 μM tyrosol and its metabolites have also been observed in TNF-α-stimulated endothelial cells [91]. It is possible that the anti-inflammatory action of tyrosol is induced through downregulation of the p38 MAPK and JNK pathways, according to a study in human peripheral blood mononuclear cells (PBMC) [92]. In phorbol-12-myristate 13-acetate (PMA)-differentiated U937 cells, 24 h of incubation with 10 μM tyrosol led to a significant attenuation of markers related to lipid oxidation [93]. Tyrosol and its acetylated at the aliphatic hydroxyl group (–OH) derivative have also been reported to inhibit PAF-induced platelet aggregation, whereas diacetylated tyrosol further induced platelet aggregation [58]. In the case of IL-1β-stimulated U937 cells, tyrosol was a more potent inhibitor of PAF-CPT at 30 min and lysoPAF-AT at 3 h than resveratrol, with its acetylated derivatives having a similar effect with the parent compound [63].

In the current study, tyrosol inhibited PAF-CPT in different concentrations; however, a more consistent low inhibition of 20% was detected in the area of 0.1 to 5 μM. The acetylation eliminated this effect, indicating that the presence of both hydroxyl-groups is needed to inhibit PAF-CPT on non-stimulated cells. Our results also demonstrated that 50 μM tyrosol, on the one hand, could inhibit PAF-CPT by approximately 44%, and on the other hand, could enhance LysoPAF-ATC and LysoPAF-ATE activity by 35% and 25%, respectively. It is noted that PAF biosynthetic pathways depend not only on the activation status of their enzymes and on the availability of their substrates but also on the energy requirement. Specifically, it has been suggested that the switch from the de novo to the remodeling pathway occurs when cellular ATP is decreased and Ca^2+^ concentration is increased [94]. Overall, acetylation weakens the phenolic potential in the case of tyrosol, and further study of the parent compound may shed light on its ability to affect the PAF biosynthetic enzymes differently via either the same or different signaling pathways.

## 4. Materials and Methods

### 4.1. Materials

The RPMI 1640 was purchased from Gibco BRL (Thermo Fisher Scientific, Waltham, MA, USA), and heat-inactivated newborn calf serum (NCS), glutamine (Glut), penicillin–streptomycin (PS), Coomassie Brilliant Blue G-250, thiazolyl blue tetrazolium bromide (MTT), and other common reagents and solvents were all obtained from Sigma (St. Louis, MO, USA). Inhibitors PD98059, SB203580, U-73122, SP600125, and Ro 31-8425·HCl, as well as lyso-PAF, free fatty acid low endotoxin bovine serum albumin (BSA), acetyl-coenzyme A, and cytidine 5′-diphosphocholine (CDP-choline), were also obtained from Sigma. 1-O-Hexadecyl-2-O-acetyl-sn-glycerol (AAG) was purchased from Enzo Life Sciences Ltd. (Farmingdale, NY, USA). PAF-18:0-d4 was obtained from Cayman Chemical (Ann Arbor, MI, USA).

### 4.2. Synthesis of the Phenolic Compounds

**Figure 5:**
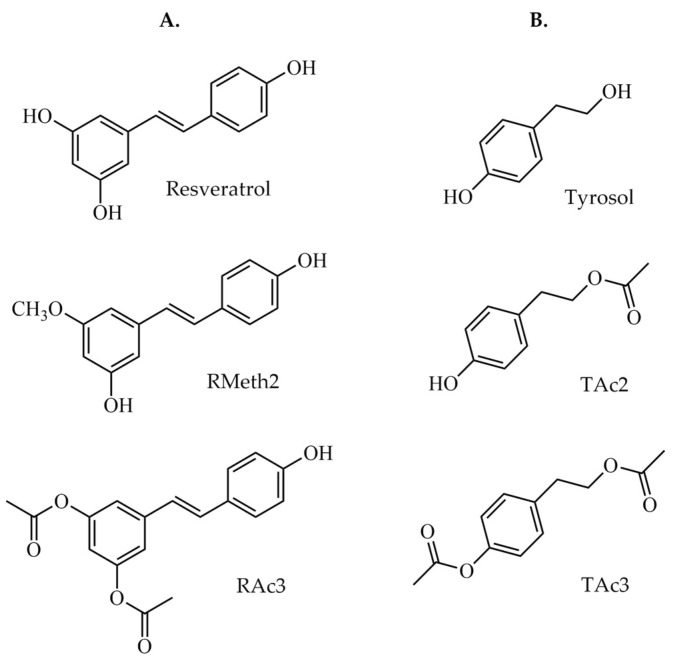
(**A**) Molecular structures of resveratrol and derivatives. (**B**) Molecular structures of tyrosol and derivatives.

Tyrosol (4-(2-hydroxyphenyl)ethanol) was purchased from Sigma. All other phenolic compounds utilized in this series of experiments were prepared at the Division of Pharmaceutical Chemistry, Department of Pharmacy, National and Kapodistrian University of Athens. Phenolics synthesis has already been described [61]. The phenolic compounds assayed in this study are trans-resveratrol (R), 3-methoxyresveratrol (RMeth2), 3,5-diacetylresveratrol (RAc3), tyrosol (T), tyrosol with the acetyl group on aliphatic oxygen (TAc2), and diacetylated tyrosol (TAc3). The molecular structures of the compounds are presented in Figure 5.

### 4.3. Cell Culture

The established human pro-monocytic cell line U937 ([95]) used in the experiments has kindly been donated by Dr. Z. Varghese, Royal Free Hospital, Centre for Nephrology, University College Medical School, London, UK. Cell culture was carried out as previously described [63] and U937 cells were synchronized in serum-free medium (SFM) for 24 h prior to any treatment with inhibitors or phenolics.

### 4.4. Cell Viability

The maximum acceptable concentrations of IL-1β, inhibitors, and phenolic compounds were identified using the MTT assay according to the Mosmann method [96] for the duration assayed in each experiment. IL-1β was dissolved in a buffer of 0.1% BSA in PBS (0.1% BSA-PBS), whereas all inhibitors and phenolic compounds were dissolved in dimethyl sulfoxide (DMSO). DMSO was calculated to be a maximum of 1% of total volume in the cell culture and was used as the negative control (vehicle) in the viability test.

### 4.5. Effect of Inhibitors on PAF Biosynthesis in Stimulated U937 Cells

Synchronized cells, at a concentration of 1.25 × 10^6^ cells/mL, were pretreated with 25 μM PD98059 (MEK 1/2 inhibitor) or 1.5 μM U-73122 (PLC-β inhibitor) for 30 min, or 20 μM SB203580 (p38 MAPK inhibitor), 5 μM SP600125 (JNK 1/2/3 inhibitor, here only in the LysoPAF-AT assay), or 2.5 μM Ro 31-8425·HCl (PKC inhibitor, here only in PAF-CPT assay) for 1 h. The inhibitor-treated cells were stimulated for 30 min for PAF-CPT or 3 h for LysoPAF-ATC in the presence of 3.3 ng/mL IL-1β, respectively. The dilution buffer 0.1% BSA-PBS was used as a negative control for IL-1β, and DMSO was used as the negative control (vehicle) for the inhibitors. The experiment was run in triplicate. The collection of the cells and the isolation of the enzyme-rich lysate has been described previously [61].

### 4.6. Effect of Resveratrol and Its Derivatives on PAF-CPT and LysoPAF-ATC

In this series of experiments, U937 cells were incubated with 10, 50, or 100 μM of either resveratrol, RMeth2, or RAc3 for 30 min, and DMSO was used as the negative control (vehicle). The short time frame was chosen to avoid transcriptional changes and to mimic the experiments on cell lysates. The experiment was run in triplicate, and cell lysates were collected as mentioned above.

### 4.7. Effect of Inhibitors on the LysoPAF-ATC Activation by Resveratrol and Its Derivatives

PD98059 25 μM, SB203580 20 μM, and U-73122 1.5 μM were used as signal transduction inhibitors. In these series of experiments, inhibitor-pretreated U937 cells were incubated with 10 or 50 μg/mL of resveratrol, RMeth2, or RAc3 for 30 min, and DMSO was used as a vehicle for both inhibitors and phenolic compounds. The experiment was run in triplicate, and cell lysates were collected as mentioned above.

### 4.8. Effect of Resveratrol, Tyrosol and Their Derivatives in U937 Cells After 24 h

The enzymes tested were PAF-CPT, LysoPAF-ATC, and LysoPAF-ATE. Cells were treated at first with resveratrol or tyrosol (0.1–100 μg/mL) as a screening experiment. To compare the derivatives with the parent compounds, resveratrol was assayed in the range of 10–100 μM, RMeth2 in the range of 5–20 μM, and RAc3 in the range of 10–75 μM. Tyrosol, TAc2, and TAc3 were assayed in the range of 0.1–5 μM. Cell treatment with the phenolic compounds was for 24 h, and DMSO was used as negative control (vehicle). The experiment was run in triplicate, and cell lysates were collected as mentioned above.

### 4.9. Enzymatic Activity Assay

The assays regarding PAF-CPT, LysoPAF-ATC, and LysoPAF-ATE have already been published [61,97]. Briefly, the protein concentration of the samples was assayed via the Bradford method [98], and sample volume containing 10 μg of protein was assayed for 5 or 10 min at 37 °C for PAF-CPT or LysoPAF-ATs, respectively, in the presence of appropriate buffers. The enzymatic reaction was stopped by adding methanol containing 2% acetic acid, and PAF was isolated via the Bligh–Dyer method [99]. Liquid chromatography–mass spectrometry (LC-MS) was used to measure the produced PAF in the presence of PAF-18:0-d4 as the internal standard [61].

### 4.10. Statistical Analysis

All results were tested for normality using the Shapiro–Wilk criterion. Normally distributed results are presented as the mean ± standard deviation (SD), whereas those not following a normal distribution are presented as the median (first quartile, third quartile). Before any comparisons, the results of each experiment were normalized as the fold difference from the baseline mean or median to avoid the fluctuation of results among experiments. For the comparison of the different cell treatments, one-way ANOVA was performed for normally distributed results, whereas Kruskal–Wallis was used for the rest. All statistical analyses were performed using GraphPad Prism, 10.2. (GraphPad Software, Boston, MA, USA).

## 5. Conclusions

Overall, we observed that IL-1β can activate PAF-CPT and LysoPAF-ATC via different signaling pathways, with PLC-β being a key enzyme in both cases. Resveratrol, 3-methoxyresveratrol, and, at a lesser extent, 3,5-diacetylresveratrol are able to also act as pro-inflammatory agents by affecting similar pathways in a distinct way, proposing a different mechanism of action between the parent compound and its derivatives. Long incubation times lead to moderate inhibition of PAF biosynthetic enzymes by the stilbene compounds, whereas tyrosolic molecules appeared to have a minimal effect on the enzymes, with the exception of tyrosol, which significantly inhibited PAF-CPT.

## Figures and Tables

**Table 1:** Viability test of phenolic compounds and inhibitors on U937 cells.

	Short-Time Effect		Long-Time Effect	
Compound	Concentration (μM)	% of Baseline	Concentration (μM)	% of Baseline
R	100	95.9 ± 9.8	100	83.8 ± 1.6
RMeth2	100	88.2 ± 3.4	20	84.2 ± 1.7
RAc3	100	98.2 ± 8.3	75	97.2 ± 2.2
T	N.A.		100	86.1 ± 5.7
TAc2	N.A.		5	88.4 ± 3.1
TAc3	N.A.		5	86.2 ± 2.2
U-73122	1.5	86.7 ± 1.5	N.A.	
PD 98059	25	96.7 ± 1.3	N.A.	
SB 203580	20	108.7 ± 2.0	N.A.	
RO-31-8425	2.5	93.6 ± 1.7	N.A.	
SP 600125	5	98.3 ± 2.5	N.A.	

Viability in the presence of the maximum tested concentration of compounds is expressed as percentage of the baseline. The percentage of the baseline is expressed as the average value ± SD. N.A., not applicable; R, resveratrol; RMeth2, 3-methoxyresveratrol; RAc3, 3,5-diacetylresveratrol; SD, standard deviation; T, tyrosol; TAc2, tyrosol acetylated at the aliphatic hydroxyl group (–OH); TAc3, diacetylated tyrosol.

**Table 2:** Effect of inhibitors on PAF-CPT activity in IL-1β-stimulated U937 cells.

PAF-CPT, IL-1β and Inhibitors, 30 min					
	IL-1β 3.3 ng/mL	U-73122 1.5 μM	PD 98059 25 μM	SB 203580 20 μM	RO-31-8425 2.5 μM
*p*-Value (vs. baseline) *	**<0.0001**	**0.0165**	**0.006**	**0.0082**	0.3836
%Change (vs. baseline)	**43.6 ± 23.5**	**21.8 ± 17.7**	**31.5 ± 22.9**	**32.2 ± 27.0**	−11.3 ± 29.6
*p*-Value (vs. IL-1β) *		**0.0155**	0.2303	0.2912	**0.0017**

* Statistically significant *p*-values (*p* < 0.05) are marked with bold. The percentage change is expressed as the average value ± SD. IL-1β, interleukin 1β; PAF-CPT, 1-alkyl-2-acetyl-sn-glycerol cholinephosphotransferase.

**Table 3:** Effect of inhibitors on LysoPAF-ATC activity in IL-1β-stimulated U937 cells.

Lyso-PAF-ATC, IL-1β and Inhibitors, 3 h					
	IL-1β 3.3 ng/mL	U-73122 1.5 μM	PD 98059 25 μM	SB 203580 20 μM	SP 600125 5 μM
*p*-Value (vs. baseline) *	**<0.0001**	0.5704	**<0.0001**	0.1741	0.8005
%Change (vs. baseline)	**23.8 ± 16.4**	−2.6 ± 10.0	**46.7 ± 13.8**	−8.3 ± 13.7	1.3 ± 10.4
*p*-Value (vs. IL-1β) *		**0.0006**	**<0.0001**	**<0.0001**	**0.0006**

* Statistically significant *p*-values (*p* < 0.05) are marked with bold. The percentage change is expressed as the average value ± SD. IL-1β, interleukin 1β; LysoPAF-ATC, Ca^2+^-dependent lysoPAF acetyltransferase.

**Table 4:** Effect of resveratrol and derivatives on PAF-CPT and LysoPAF-ATC in U937 cells after 30 min.

		PAF-CPT		LysoPAF-ATC	
Phenolic Compound		%Change	*p*-Value *	%Change	*p*-Value *
R	100 μM	−11.7 ± 11.4	0.160	−17.8 ± 7.2	0.1313
R	50 μM	−2.2 ± 14.4	0.774	**14.5 ± 19.5**	**0.0424**
R	10 μM	8.4 ± 18.0	0.257	**35.0** **± 16.4**	**<0.0001**
RMeth2	100 μM	3.0 ± 8.8	0.682	**44.8 ± 12.7**	**<0.0001**
RMeth2	50 μM	−0.1 ± 15.8	0.992	**28.7 ± 24.3**	**0.0001**
RMeth2	10 μM	7.9 ± 7.8	0.289	13.0 ± 22.1	0.0682
RAc3	100 μM	3.9 ± 14.7	0.600	**28.0 ± 11.7**	**0.0058**
RAc3	50 μM	−8.2 ± 16.0	0.268	10.1 ± 17.3	0.1679
RAc3	10 μM	0.9 ± 10.6	0.902	−3.4 ± 37.4	0.6409

* Statistically significant *p*-values (*p* < 0.05) are marked with bold. The percentage change is expressed as the average value ± SD.

**Table 5:** Effect of 25 μM PD 98059 on resveratrol, RMeth2, and RAc3’s effect on LysoPAF-ATC.

Compared to	Baseline		Phenolic Compound	
	%Change	*p*-Value *	%Change	*p*-Value *
R 50 μΜ	**14.5 ± 19.5**	**0.0413**		
R 50 μM + PD 98059	12.4 ± 17.4	0.2454	−1.8 ± 15.2	0.8503
R 10 μM	**35.0 ± 16.4**	**<0.0001**		
R 10 μM + PD 98059	1.5 ± 15.0	0.8887	**−24.8 ± 11.1**	**0.0037**
RMeth2 50 μM	**28.7 ± 24.3**	**<0.0001**		
RMeth2 50 μM +PD 98059	**40.8 ± 27.1**	**<0.0001**	9.4 ± 21.0	0.2375
RMeth2 10 μM	13.0 ± 22.1	0.0669		
RMeth2 10 μM + PD 98059	**49.3 ± 10.7**	**<0.0001**	**32.1 ± 9.5**	**0.0011**
RAc3 50 μM	10.1 ± 17.3	0.1660		
RAc3 50 μM + PD 98059	1.7 ± 14.0	0.8657	−7.7 ± 12.7	0.4128
RAc3 10 μM	−3.4 ± 37.4	0.6395		
RAc3 10 μM + PD 98059	**31.0 ± 7.9**	**0.0022**	**35.6 ± 8.2**	**0.0011**

* Statistically significant *p*-values (*p* < 0.05) are marked with bold. The percentage change is expressed as the average value ± SD.

**Table 6:** Effect of 1.5 μM U-73122 on resveratrol, RMeth2, and RAc3’s effect on LysoPAF-ATC.

Compared to	Baseline		Phenolic Compound	
	%Change	*p*-Value *	%Change	*p*-Value *
R 50 μΜ	**14.5 ± 19.5**	**0.0402**		
R 50 μM + U-73122	**39.9 ± 20.5**	**<0.0001**	**22.2 ± 17.9**	**0.0127**
R 10 μM	**35.0 ± 16.4**	**<0.0001**		
R 10 μM + U-73122	**44.0 ± 24.1**	**0.0002**	6.7 ± 17.9	0.4646
RMeth2 50 μM	**28.7 ± 24.3**	**<0.0001**		
RMeth2 50 μM + U-73122	**32.1 ± 16.2**	**0.0014**	2.6 ± 12.6	0.7398
RMeth2 10 μM	13.0 ± 22.1	0.0654		
RMeth2 10 μM + U-73122	**21.9 ± 14.8**	**0.0402**	7.9 ± 13.1	0.4074
RAc3 50 μM	10.1 ± 17.3	0.1636		
RAc3 50 μM + U-73122	**24.2 ± 14.4**	**0.0151**	12.8 ± 13.1	0.1696
RAc3 10 μM	−3.4 ± 37.4	0.6376		
RAc3 10 μM + U-73122	−3.0 ± 8.5	0.7816	0.5 ± 8.8	0.9656

* Statistically significant *p*-values (*p* < 0.05) are marked with bold. The percentage change is expressed as the average value ± SD.

**Table 7:** Effect of 20 μM SB 203580 on resveratrol, RMeth2, and RAc3’s effect on LysoPAF-ATC.

Compared to	Baseline		Phenolic Compound	
	%Change	*p*-Value *	%Change	*p*-Value *
R 50 μΜ	**14.5 ± 19.5**	**0.0404**		
R 50 μM + SB 203580	**−25.8 ± 9.6**	**0.0165**	**−35.2 ± 8.4**	**0.0003**
R 10 μM	**35.0 ± 16.4**	**<0.0001**		
R 10 μM + SB 203580	**−49.1 ± 8.3**	**<0.0001**	**−62.3 ± 6.2**	**<0.0001**
RMeth2 50 μM	**28.7 ± 24.3**	**<0.0001**		
RMeth2 50 μM + SB 203580	**−21.5 ± 16.7**	**0.0444**	**−39.0 ± 13.0**	**<0.0001**
RMeth2 10 μM	13.0 ± 22.1	0.0656		
RMeth2 10 μM + SB 203580	−7.3 ± 22.4	0.4619	**−18.0 ± 19.9**	**0.0461**
RAc3 50 μM	10.1 ± 17.3	0.1639		
RAc3 50 μM + SB 203580	−20.1 ± 18.1	0.0604	**−27.5 ± 16.5**	**0.0066**
RAc3 10 μM	−3.4 ± 37.4	0.6378		
RAc3 10 μM + SB 203580	**−23.7 ± 4.1**	**0.0435**	−21.0 ± 4.3	0.0918

* Statistically significant *p*-values (*p* < 0.05) are marked with bold. The percentage change is expressed as the average value ± SD.

**Table 8:** Effect of resveratrol and derivatives on PAF biosynthetic enzymes in U937 cells after 24 h.

Phenolic Compound		PAF-CPT		LysoPAF-ATC		LysoPAF-ATE	
		%Change	*p*-Value *	%Change	*p*-Value *	%Change	*p*-Value *
R	100 μM	**−14.8** **(−31.3, −7.6)**	**0.0004**	**−43.4** **(−47.7, −33.9)**	**<0.0001**	**−41.6** **(−48.3, −28.2)**	**<0.0001**
R	50 μM	**−26.7** **(−44.9, −26.7)**	**0.0005**	**−24.0** **(−38.6, −18.5)**	**<0.0001**	**−32.6** **(−39.3, −25.8)**	**<0.0001**
R	10 μM	**−12.7** **(−26.7, 7.2)**	**0.0105**	−5.4 (−21.3, 23.5)	0.5838	0.6 (−28.6, 11.0)	0.3062
R	5 μM	−8.5 (−13.8, −3.0)	0.1289	17.6 (4.4, 26.1)	0.0624	7.1 (−4.5, 7.6)	0.5607
R	1 μM	3.4 (−2.4, 8.9)	0.6101	1.6 (−6.9, 14.0)	0.6932	17.9 (−7.9, 26.2)	0.2139
R	0.1 μM	−10.1 (−18.1, −1.4)	0.0702	4.6 (−3.6, 17.4)	0.418	14.1 (−12.2, 26.2)	0.3424
RMeth2	20 μM	**−39.3** **(−47.2, −33.7)**	**<0.0001**	12.7 (−3.7, 14.2)	0.8145	**−21.0** **(−23.7, −18.5)**	**0.0035**
RMeth2	10 μM	**−20.2** **(−40.9, −6.9)**	**0.0005**	**−36.3** **(−51.3, −26.1)**	**<0.0001**	−13.5 (−21.9, −3.8)	0.1534
RMeth	5 μM	**−15.0** **(−17.9, −5.4)**	**0.0269**	**−11.1** **(−24.0, −4.5)**	**0.0366**	1.1 (−5.3, 13.4)	0.5612
RAc3	75 μM	**−14.7** **(−64.1, −10.0)**	**<0.0001**	**−23.0** **(−32.6, −14.7)**	**0.0028**	−10.8 (−23.0, −9.1)	0.0936
RAc3	50 μM	**−26.7** **(−37.1, −21.5)**	**<0.0001**	3.2 (−4.7, 11.9)	0.7164	−1.3 (−9.6, 8.3)	0.7816
RAc3	10 μM	**−24.2** **(−33.6, −3.9)**	**0.0005**	4.1 (−10.1, 22.5)	0.701	7.1 (−15.5, 25.0)	0.4637

* Statistically significant *p*-values (*p* < 0.05) are marked with bold. The percentage change is expressed as the median (first quartile, third quartile). LysoPAF-ATE, Ca^2+^-independent lysoPAF acetyltransferase

**Table 9:** Effect of tyrosol and derivatives on PAF biosynthetic enzymes in U937 cells after 24 h.

Phenolic Compound		PAF-CPT		LysoPAF-ATC		LysoPAF-ATE	
		%Change	*p*-Value *	%Change	*p*-Value *	%Change	*p*-Value *
T	100 μM	8.7 (−2.5, 18.4)	0.2891	**−11.8** **(−20.9, 3.9)**	**0.0193**	8.3 (−17.5, 17.9)	0.5867
T	50 μM	**−44.7** **(−48.2, −23.2)**	**0.0005**	**44.1** **(35.3, 52.5)**	**0.002**	**32.4** **(16.2, 39.2)**	**0.003**
T	10 μM	−9.0 (−19.8, 6.6)	0.1597	−1.1 (−7.4, 12.8)	0.4879	0.0 (−17.9, 28.2)	0.56
T	5 μM	**−16.3** **(−36.6, 7.1)**	**0.0238**	6.8 (0.8, 13.1)	0.1201	3.3 (−14.9, 8.9)	0.9267
T	1 μM	**−16.1** **(−29.4, −3.0)**	**0.0015**	3.3 (−7.9, 14.7)	0.3912	4.1 (−6.3, 13.0)	0.3321
T	0.1 μM	**−23.7** **(−30.7, −10.1)**	**0.0001**	4.4 (−11.7, 20.1)	0.3465	10.8 (−8.9, 27.0)	0.1117
TAc2	5 μM	3.5 (−14.8, 17.3)	0.7534	**−13.6** **(−18.6, −4.2)**	**0.0179**	−6.6 (−14.4, 5.9)	0.2404
TAc2	1 μM	2.3 (−18.2, 30.2)	0.9294	3.8 (−3.6, 20.1)	0.228	5.4 (−8.9, 14.6)	0.3435
TAc2	0.1 μM	13.1 (−18.1, 26.7)	0.7818	−2.1 (−8.3, 2.2)	0.8988	5.8 (−11.3, 11.4)	0.5235
TAc3	5 μM	−2.9 (19.8, 16.2)	0.3879	1.9 (−15.7, 12.7)	0.77	−9.0 (−13.8, 3.2)	0.3635
TAc3	1 μM	−11.3 (−26.3, 7.7)	0.0806	−8.5 (−17.0, 7.9)	0.14	**−21.8** **(−29.3, −1.3)**	**0.0027**
TAc3	0.1 μM	**−13.0** **−34.5, 8.2)**	**0.0491**	−9.8 (−19.9, 4.5)	0.1397	−0.2 (−6.5, 6.5)	0.8604

* Statistically significant *p*-values (*p* < 0.05) are marked with bold. The percentage change is expressed as the median (first quartile, third quartile).

## Data Availability

The data of the present study are available from the corresponding author upon request.
